# Evidence of Clinical and Laboratory Correlation of Itraconazole Resistance in *Sporothrix brasiliensis* Infection: Case Report

**DOI:** 10.3390/microorganisms12112132

**Published:** 2024-10-24

**Authors:** John Verrinder Veasey, Ana Paula Carvalho Reis, Giovanna Azevedo Celestrino, Camila Estacia Silva, Eduarda Souza Santos, Denise Polizel Mendes, Tania Sueli Andrade, Lucas Xavier Bonfietti, Gil Benard, Maria Glória Texeira Sousa

**Affiliations:** 1 School of Medical Sciences, Santa Casa de Sao Paulo, São Paulo 01221010, Brazil; johnvveasey@gmail.com; 2 Laboratory of Medical Mycology LIM-53, Clinical Dermatology Division, Hospital das Clínicas FMUSP, Faculdade de Medicina FMUSP, Institute of Tropical Medicine, University of São Paulo, São Paulo 05403000, Brazil; aninhareis97@usp.br (A.P.C.R.); acgiovanna@usp.br (G.A.C.); camilaestacia@usp.br (C.E.S.); eduardassantos1803@gmail.com (E.S.S.); denisepolizel@hotmail.com (D.P.M.); bengil60@gmail.com (G.B.); 3 Department of Culture Collection, Adolfo Lutz Institute, Secretary of Health, Sao Paulo 01246000, Brazil; 4 Health Department, Mycology Nucleus of Instituto Adolfo Lutz, São Paulo 01246000, Brazil; lucasxbonfietti@yahoo.com.br

**Keywords:** antifungal resistance, Sporotrichosis, itraconazole, *Sporothrix brasiliensis*

## Abstract

Sporotrichosis is a subcutaneous infection caused by fungi from the genus *Sporothrix*, among which *Sporothrix brasiliensis* displays high virulence and transmissibility. So far, classical antifungal agents have been efficient against *S. brasiliensis*, but here we describe the first case of therapeutic failure and a high minimum inhibitory concentration (MIC) in relation to itraconazole.

## 1. Introduction

An 83-year-old male, with a history of type 2 diabetes and chronic obstructive pulmonary disease (COPD), presented at our clinic with a one-month history of cutaneous lesions on the right arm after having been bitten by his cat.

## 2. Detailed Case Description

**Figure 1:**
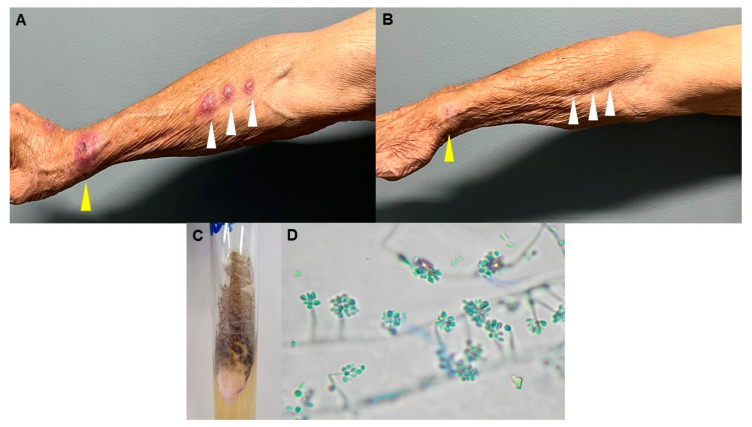
Clinical presentation and mycological profile. (**A**) Picture of the lesions on the patient’s forearm taken after 4 months of treatment with ITZ 100 mg/day and (**B**) after 6 months of treatment with KI 3.5 g/day. The yellow arrow indicates the original lesion site, and the white arrows point to the nodules developed afterwards. (**C**) Growth of *S. brasiliensis* isolated from the lesion; off-white to cream color colonies with raised centers appeared after 7 days of incubation at 28 °C on Sabouraud agar. (**D**) Micromorphology of the sympodial and sessile conidia of *S. brasiliensis* recovered from the culture in Sabouraud agar after 12 days of incubation at 28 °C. Hyaline conidia sympodially arranged in a bouquet-like fashion at the ends of the conidiophores compatible with *Sporothrix* spp. were visualized, magnification ×400.

The patient, the owner of three cats, lived in the city of São Paulo, Brazil. He reported being bitten on the right wrist by one of them, and a few days later, he noted the appearance of an ulcerated lesion on the bitten area followed by the development of small nodules on the right forearm, reaching the elbow. The patient consulted different physicians, having been diagnosed with a presumed bacterial infection and treated unsuccessfully with clindamycin, cefuroxime, and amoxycillin/clavulanic acid via the oral route. He occasionally complained of chills but did not report fever. On admission at our dermatology facility, he presented with an ulcerated lesion on the right wrist with discrete thick purulent discharge and three painful nodules of around 1 cm each, following the lymphatic pathway towards the elbow (Figure 1A).

Lymphocutaneous sporotrichosis was our main diagnostic hypothesis due to the typical clinical characteristics in association with the history of zoonotic exposure in an epidemic area. The metropolitan area of São Paulo has been undergoing an outbreak of feline sporotrichosis since 2013, followed by an outbreak of human sporotrichosis since 2015 [1].

A biopsy of one of the nodules in the forearm was performed and sent for histopathological examination and mycological culture. Histopathological analysis showed a chronic inflammatory process with a microabscess and, after periodic acid–Schiff (PAS) and Grocott staining, numerous rounded yeasts compatible with *Sporothrix spp* were observed. The fungal culture yielded white to cream-colored filamentous colonies, which over time became darkish in the center (Figure 1C). Microscopic examination revealed hyaline hyphae with conidiophores producing oval to round hyaline conidia at the tip, reinforcing our hypothesis (Figure 1D). The isolate was further identified by amplifying a segment of the calmodulin (CAL) gene with PCR using the primers CALF (5′-AAGGACGGCGATGGTTAGTCA-3′) and CALR (5′-AGCCGAGATGAAGCCGTTGTT-3′) and performing Sanger sequencing in an ABI PRISM^®^ 3500 Genetic Analyzer, Applied Biosystems, Foster City, CA, USA. Initial nucleotide BLAST analysis was performed on the NCBI database that showed *Sporothrix brasiliensis* as the closest BLAST match (GenBank accession number PQ383413) [2].

The patient was initially prescribed a 5-month course of itraconazole (ITZ, 100 mg daily). However, after two returns within 4 months of follow up, no remarkable improvement of the cutaneous lesions was observed, raising the concern of drug resistance. We thus performed an antifungal susceptibility test of the isolate. The antifungal drugs used in this study were itraconazole, voriconazole terbinafine, and amphotericin B (all purchased from Sigma-Aldrich, Burlington, MA, USA). According to EUCAST document E.DEF 9.4, we carried out the broth dilution method with adaptations for the slower-growing dimorphic fungus *Sporothrix* sp. The suspension of each conidial inoculum was prepared from 7-day-old cultures at 28 °C on potato dextrose agar slants by adding 5 mL of sterile water with 0.1% Tween 20 and carefully rubbing the conidia with a sterile cotton swab. We adjusted the suspension in a hemocytometer chamber. The inoculum (100 µL) was dispensed into microdilution wells containing the drug, reaching a final density of 1 to 2 × 10^5^ CFU/mL. After that, we incubated the plates at 35 °C and read them at 48 h. The minimum inhibitory concentrations (MICs) were determined by means of visual inspection of complete growth inhibition, except for terbinafine, whose MIC was considered as the one that inhibited approximately 90% of fungal growth compared to the controls. All the runs included *Candida krusei* ATCC 6258 and *Candida parapsilosis* ATCC 22019 as controls [3,4,5].

The isolate’s MICs were as follows: ITZ, >8 mg/L; TRB, 0.12 mg/L; posaconazole, 0.5 mg/L; amphotericin B, 0.5 mg/L; and voriconazole, >8 mg/L. The results for the quality control strains were within the recommended limits.

The confirmation of therapeutic failure and high MICs in relation to ITZ prompted its replacement with potassium iodide (KI, 35 drops twice daily, totaling 3.5 g/day) associated with cryotherapy (twice a week). Under this new protocol, the lesions progressively subsided, and the treatment was stopped after 6 months with complete clinical resolution (Figure 1B). The patient did not report any of the side effects occasionally related to the oral intake of KI, such as gastrointestinal manifestations. No relapse was diagnosed after one year of follow up.

## 3. Discussion

In this case report, we presented evidence of a clinical and laboratory correlation of resistance to ITZ in a sporotrichosis case caused by *S. brasiliensis*. The failure of the oral administration of ITZ to ameliorate the lesions after 4 months of treatment was solved via the initiation of KI treatment and cryotherapy, yielding marked clinical improvement and being able to completely eradicate the fungus after 5 months of treatment. To the best of our knowledge, this is the first description of therapeutic failure associated with a high MIC in relation to ITZ in a human patient infected with *S. brasiliensis*.

ITZ is the drug of choice for human and feline sporotrichosis caused by *S. brasiliensis*, according to guidelines from the Brazilian Society of Dermatology. TRB and KI stay as second options, also with high efficacy [6].

There are only a few descriptions of sporotrichosis patients who were refractory to conventional treatments, but no correlation has been found between the antifungal resistance of the isolates and the clinical outcome or treatment response. Lyra et al. (2021), for example, reported a patient with fixed cutaneous sporotrichosis refractory to ITZ and TRB who achieved resolution only after 2 months of treatment with KI. However, in vitro testing of the patient’s isolate still indicated susceptibility to both ITZ and TRB [7]. In 2017, two studies proposed the epidemiological cut-off of 2 µg/mL for ITZ for both *S. schenckii* and *S. brasiliensis* [8,9], which has since been used as a parameter to interpret results from *Sporothrix* spp. susceptibility tests. While some studies found very low prevalence of resistant (non-wild type) isolates to both ITZ and TRB, ranging from 0% to ≤5% of the non-wild type isolates [10,11], others reported ≥20% of the isolates with high MICs (≥4 μg/mL) in relation to ITZ [12].

The observed differences in the MIC results and the proportion of wildtype and non-wildtype isolates could be explained by the methodological heterogeneity among laboratories, which highlights the challenge posed by antifungal susceptibility test studies regarding reproducibility and translation to clinical practice. Albeit not always straightforward, it is conceivable to assume that isolates that exhibit a high MIC in relation to ITR and TRB would translate into treatment failure. Therefore, the emergence of non-wildtype *S. schenckii* and *S. brasiliensis* isolates in relation to ITZ, TRB, amphotericin B, and voriconazole points towards a concerning scenario of antifungal therapy failure with these compounds. Mechanistically, prolonged exposure to antifungals and host immunity could exert selective pressure on *S. brasiliensis*, favoring the development of resistance phenotypes such as increased melanin production, low genetic diversity, and mutations in cytochrome P450 [13].

Even though the clinical impact of the emergence of in vitro resistance in *S. brasiliensis* remains unclear and has yet to be established, the reports of treatment failure, as seen in our case report here, underscores the necessity for closer epidemiological surveillance, particularly of patients and animals who present with a “slow” or atypical response to treatment, and those with severe forms who require prolonged treatments. In parallel, intense efforts to isolate and determine the susceptibility profile of the etiological agent are becoming mandatory to better clarify this scenario of antifungal resistance.

Specifically in the current epidemic outbreak of sporotrichosis ongoing in Brazil and spreading throughout Latin America, the emergence of resistant isolates potentiates the public health threat caused by zoonotic sporotrichosis, already showing worrying proportions due to the increasing number of persons and animals affected without accessible and effective control measures, the lack of early diagnosis, and the poor treatment of susceptible hosts [14].

## 4. Conclusions

In conclusion, we report original data showing therapeutic failure and a high MIC in relation to ITZ in the treatment of *S. brasiliensis*. We emphasize the urgent need for closer surveillance of the emergence of antifungal resistance and antifungal stewardship to help to control this public health threat.

## Data Availability

The original contributions presented in the study are included in the article, further inquiries can be directed to the corresponding author.
